# Supplementary material for: Increase in Virus-Specific Mucosal Antibodies in the Upper Respiratory Tract Following Intramuscular Vaccination of Previously Exposed Horses Against Equine Herpesvirus Type-1/4
Source: Vaccines (Basel). 2025 Mar 10;13(3):290. doi: 10.3390/vaccines13030290 (PMC11946061; doi:10.3390/vaccines13030290)
Supplement: Supplementary file 1 [file vaccines-13-00290-s001.zip › Suppl Figure S1 & S2.pptx]

## Slide 1
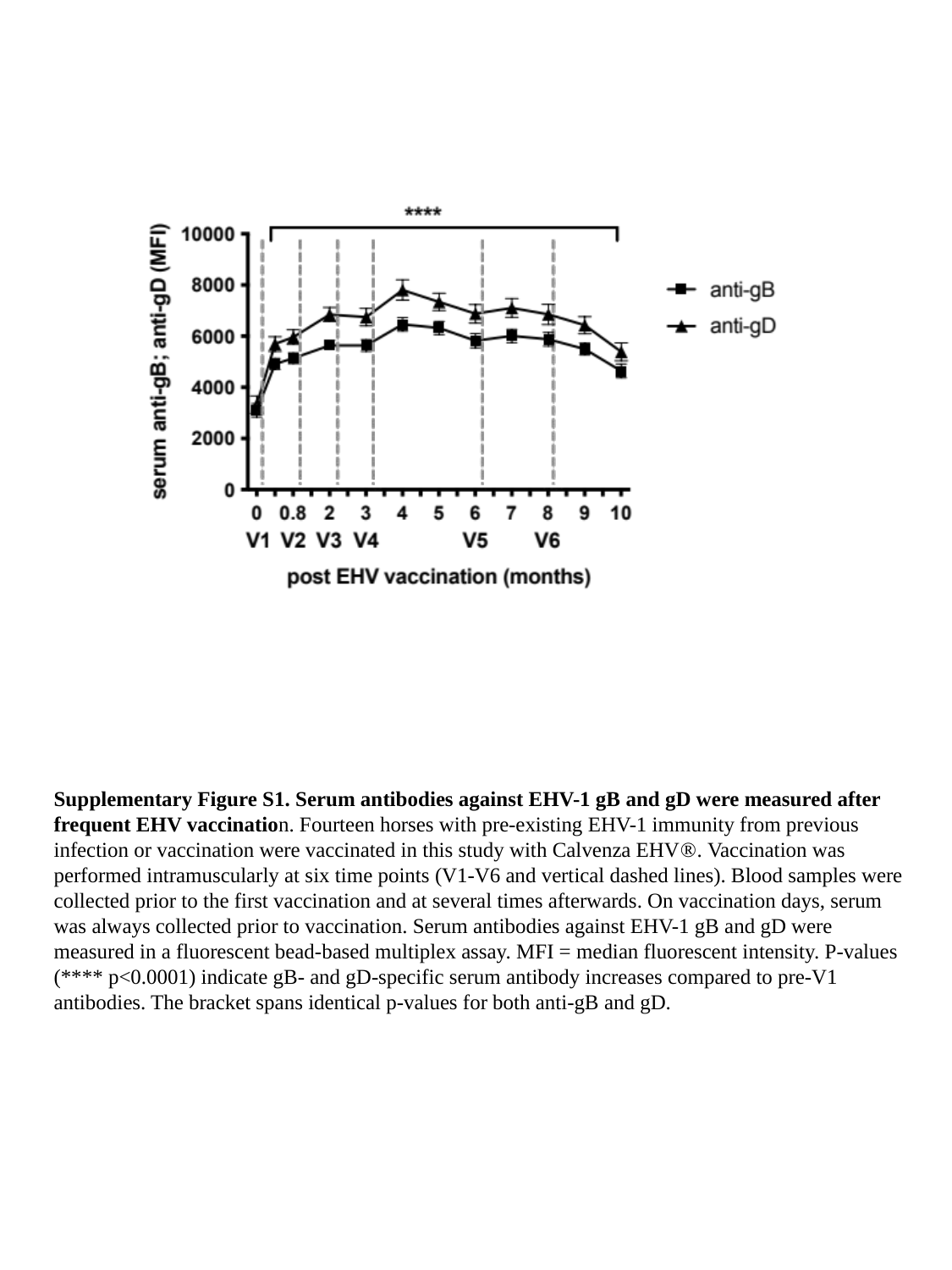

Supplementary Figure S1. Serum antibodies against EHV-1 gB and gD were measured after frequent EHV vaccination. Fourteen horses with pre-existing EHV-1 immunity from previous infection or vaccination were vaccinated in this study with Calvenza EHV. Vaccination was performed intramuscularly at six time points (V1-V6 and vertical dashed lines). Blood samples were collected prior to the first vaccination and at several times afterwards. On vaccination days, serum was always collected prior to vaccination. Serum antibodies against EHV-1 gB and gD were measured in a fluorescent bead-based multiplex assay. MFI = median fluorescent intensity. P-values (**** p<0.0001) indicate gB- and gD-specific serum antibody increases compared to pre-V1 antibodies. The bracket spans identical p-values for both anti-gB and gD.

## Slide 2
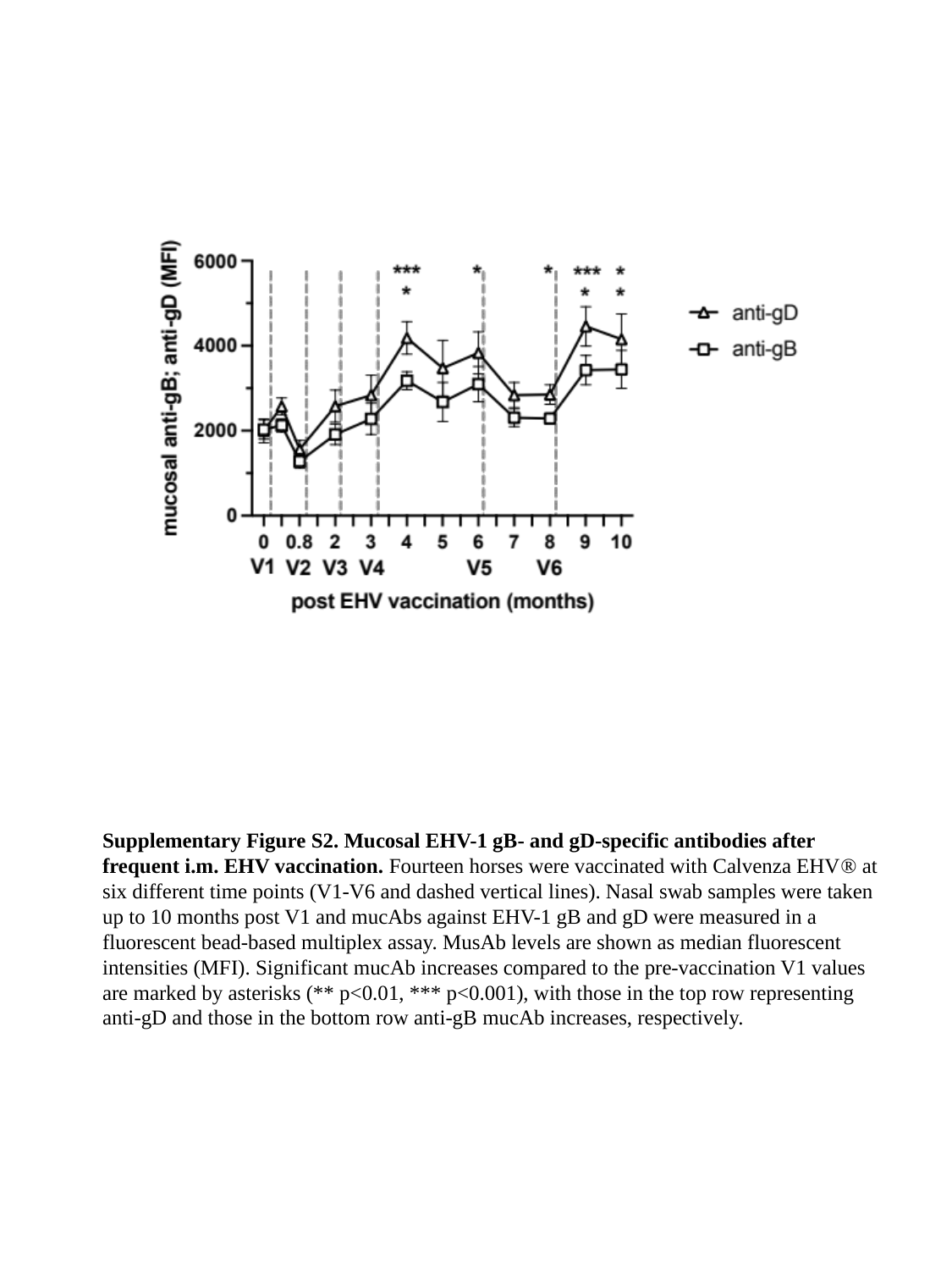

Supplementary Figure S2. Mucosal EHV-1 gB- and gD-specific antibodies after frequent i.m. EHV vaccination. Fourteen horses were vaccinated with Calvenza EHV at six different time points (V1-V6 and dashed vertical lines). Nasal swab samples were taken up to 10 months post V1 and mucAbs against EHV-1 gB and gD were measured in a fluorescent bead-based multiplex assay. MusAb levels are shown as median fluorescent intensities (MFI). Significant mucAb increases compared to the pre-vaccination V1 values are marked by asterisks (** p<0.01, *** p<0.001), with those in the top row representing anti-gD and those in the bottom row anti-gB mucAb increases, respectively.
